# Effects of Neuropeptide Substance P on Proliferation and β-Cell Differentiation of Adult Pancreatic Ductal Cells

**DOI:** 10.3389/fnins.2018.00806

**Published:** 2018-11-05

**Authors:** Nan Zhang, Di Gao, Yudan Liu, Sihan Ji, Lei Sha

**Affiliations:** Department of Neuroendocrine Pharmacology, School of Pharmacy, China Medical University, Shenyang, China

**Keywords:** Substance P, calcitonin gene-related peptide, pancreatic ductal cells, proliferation, β-cell differentiation, NK-1 receptor

## Abstract

**Purpose:** The pancreas is innervated by sensory nerves, parasympathetic and sympathetic nerves. The classical neurotransmitters, acetylcholine and noradrenaline, and some kind of neuropeptides are contained in the terminals of these nerves. Neuropeptides substance P (SP) and calcitonin gene-related peptide (CGRP) co-released from the primary sensory fibers have been identified as the key neurotransmitters in pancreas. Pancreatic ductal epithelium cells are one of the important sources of the pancreatic islet β-cell neogenesis. We hypothesized that SP and CGRP might play a role on proliferation of ductal cells and differentiation of ductal cells toward the β-cell neogenesis.

**Methods:** Primary ductal cells of rat pancreas at the third passage (P3) were used. The identification of P3 cells were confirmed with flow cytometry analysis and immunostaining by CK19 (the ductal cell marker). Proliferation of ductal cells was verified by CCK-8 assay and Ki67 immunostaining. Differentiation of ductal cells was determined with immunostaining and flow cytometry. Possible mechanism was explored by testing the key proteins of Wnt signaling using Western blot analysis.

**Results:** Our data showed that SP but not CGRP promoted proliferation of ductal cells. Moreover, NK-1 receptor antagonist L-703,606 blocked the SP-induced stimulation of proliferation. The results of Western blot analysis showed that L-703,606 attenuated the effects of substance P on NK1R, GSK-3β, and β-catenin expression. However, SP did not directly induce the differentiation of ductal cells into β-cells, and did not promote the progression of ductal cells to differentiate into more insulin-produced cells in induction medium.

**Conclusions:** These findings suggested that SP but not CGRP promoted proliferation of adult pancreatic ductal cells. SP promoted proliferation of ductal cells but not differentiation into β-cells. NK1R and Wnt signaling pathway might be involved in the mechanism of promoting the proliferation of ductal cells by SP. Findings in this study indicated the lack of SP might be a possible indicator for the initial of diabetes. SP could also be used as a drug candidate for the treatment of diabetes.

## Introduction

The β-cell mass is dynamic and is regulated to maintain glucose homeostasis in response to the changes in physiological and pathological demands (Bonner-Weir, 2000; Halban et al., 2010; Juhl et al., 2010). When the demand for insulin is increased, the β-cell mass is expanded by replication and/or hypertrophy of pre-existing β-cells (Dor, 2006), and β-cell neogenesis from pancreatic progenitor cells located in duct epithelial cells or surrounding centroacinar cells/terminal ductules (Bonner-Weir et al., 2010). Therefore, strategies targeted to improve proliferation or differentiation of ductal cells may lead to improvements in the alleviation of β-cell loss pathology.

Pancreatic ductal cells are a part of exocrine component of pancreas and can act as pancreatic stem cells and the progenitor cells of islet β cells. In injured adult mouse pancreas, β-cell progenitors located in the ductal lining were activated (Xu et al., 2008). Several *in vitro* studies have shown that adult pancreatic ductal cells can differentiate into insulin-producing cells (Fukazawa et al., 2006; Seeberger et al., 2006; Li et al., 2011). Proliferating pancreatic ductal epithelium cells were induced to differentiate into β-cells with TNF-like weak inducer of apoptosis (Wu et al., 2013). Expanded pancreatic ductal cells also differentiated into insulin-producing β-cells in an appropriate environment (Rovira et al., 2010). Capacity of self-renewal and pluripotency is an important feature of stem cells. Despite the differentiation capability of ductal cells has been demonstrated, the proliferation potential and the possible factors controlling of growth in these cells is not well-understood.

The importance of the nervous system in maintaining body homeostasis has previously been described, and it is suggested that organogenesis and tissue repair are under neural control (Besedovsky and del Rey, 1996). There is increasing evidence that neuroendocrine-remodeling does take place in the pancreatic islets of diabetic disease models (Persson-Sjögren et al., 2005; Razavi et al., 2006). Two neuropeptide substance P (SP) and calcitonin gene-related peptide (CGRP) have been found to tightly link to the development of diabetes. SP mediates insulin secretion and plays an important role in the development of type I diabetes (Razavi et al., 2006). CGRP is also involved in the activity of insulin secretion and contributes to the development of type II diabetes (Gram et al., 2007; Tanaka et al., 2011). SP and CGRP fibers not only innervate islets, but also innervate pancreatic ducts (Razavi et al., 2006; Gram et al., 2007), suggesting that SP and CGRP might modulate the activity of pancreatic ducts. We hypothesized that the innervations of the primary sensory fibers to the pancreatic ducts play a role on ductal epithelium cells proliferation and differentiation toward the β-cell neogenesis.

In the present study we investigated the effects of SP and CGRP on primary cultured ductal cells of rat pancreas. We examined the effects of SP and CGRP on proliferation of pancreatic ductal cells, and further the effect of SP on differentiation of ductal cells toward β-cells. Moreover, we investigated the possible mechanism of the proliferative promotion effects of SP.

## Materials and methods

### Animals

Sprague Dawley rats (male, 2 months old) were purchased from the Animal Center of China Medical University. All animal protocols were approved by the Animal Care Commitee in China Medical University (Shenyang, China) and performed according to institutional guidelines.

### Preparation of substance P (SP) and calcitonin gene related peptide (CGRP)

SP was purchased from Millipore Co. (Catalog number: 05-23-0600-1MGCN, Billerica, MA, USA), CGRP was gained from Bioss Co. (Catalog number: bs-0791P, Beijing, China), and both kept protected from light during the experiments. Stock solution of SP (1 mg/ml) and CGRP (1 mg/ml) were dissolved in distilled water and stored at −20°C.

### Primary culture and identification of pancreatic ductal cells

Adult Sprague Dawley rats were sacrificed and the pancreases were rapidly removed. Pancreases were then dissociated with V collagenase (1.5 g/L). The digested tissues were triturated through 600 μm cell strainer to obtain primary ductal cells. The cultures were grown in the complete medium containing DMEM/F12 supplemented with 10% fetal bovine serum (FBS), 100 U/ml Penicillin and 100 μg/ml Streptomycin (all from Gibco) at 37°C in a humidified atmosphere with 5% CO_2_. The medium was changed 24 h after, and the non-adherent cells were discarded. The attached cells were labeled P0, and the medium was changed again every 3 days. When cells became 90% confluent, cultures were dissociated with trypsin (1 ml 0.25% trypsin) at 37°C 2–3 min, and serum was used to inactivate the enzyme. Cells were then expanded at the third passage (P3) for purification. Cells were characterized for the ductal cell marker cytokeratin19 (CK19) by immunocytochemistry and flow cytometry.

Cells were plated at a density of 1.0 × 10^5^ cells/ml and cultured. At day 1, 3, 5, 7, and 9. Cells in each well were collected and counted by hemocytometer. At least 6 wells were assessed at each time point. Growth curve was designed to reveal the self-renewal capacity of the cells.

### Assay for proliferation of ductal cells

Disassociated ductal cells at 3rd passage (P3) were plated in 96 well-plates at a density of around 1.0 × 10^5^ cells per well. The cells were divided into groups randomly and treated with saline (1 mM) or serial concentrations of SP (10^−6^, 10^−5^, 10^−4^, 10^−3^, 10^−2^, 10^−1^, 1, and 10 μM; *n* = 6 wells for each) or CGRP (10^−6^, 10^−5^, 10^−4^, 10^−3^, 10^−2^, 10^−1^, 1, and 10 μM; *n* = 6 wells for each) for 3 days. In the other experiment, cells were treated with SP or SP plus L-703,606 (1, 2, 4, 6, 8, 10 μM) for 3 days. The medium containing SP or CGRP was changed every 24 h to ensure the concentration of neuropeptides in the medium. After treatments, cell viability was determined by CCK-8 test. Briefly, the cells were incubated with 10 μl CCK-8 (Invitrogen) at 37°C for 4 h. Absorbance at 490 nm was determined with a microplate reader (Thermo Fisher Scientific Inc., USA). The results were presented as percentages of the value of normal control cells.

The proliferation potential of ductal cells was tested by Ki67 immunostaining after treated with serial doses of SP (10^−3^, 10^−2^, 10^−1^, and 1 μM *n* = 6 wells for each) with or without L-703,606 (2 μM) for 3 days. The medium containing SP was changed every 24 h. Cells were fixed with 4% paraformaldehyde and then incubated with primary antibody at 4°C overnight. The primary antibodies used were as follows: mouse anti CK19 (1:100; Proteintech, Wuhan, China) and rabbit anti Ki67 (1:50, Abcam, Cambridge, UK), rabbit anti NK1R (1:100) and mouse anti Ki67 (1:100, All from Bioss, Beijing, China). After rinsing with PBS, cells were incubated with Cy3 or FITC-conjugated secondary antibodies (1:200; all from Jackson ImmunoResearch Lab, West GroveCity, PA, USA) at room temperature for 1 h. Immunofluorescence controls were performed without primary antibodies. The cells were covered with mounting medium (Vector Laboratories, Burlingame, CA, USA) containing DAPI (0.5 μg/mL; Sigma, St. Louis, MO, USA). Results were visualized with fluorescence microscopy (Nikon Eclipse E600; Nikon Corporation, Tokyo, Japan). Cells were counted in triplicate cultures in ten randomly chosen areas under the microscope. NIH ImageJ was used to count Ki67^+^/CK19^+^ cells, and the mean numbers were used for analysis.

### Detection of differentiation and immunocytochemistry

P3 ductal cells were directly treated with SP (10^−2^, 10^−1^, and 1 μM) or treated with SP and insulin-produced cell induction medium (DMEM/F12 plus 2% FBS, 10 mmol/L Niacinamide, 20 ng/ml HGF, 20 μg/L bFGF, and 10 nmol/L exendin-4) for 28 days and immunostained with CK19 and Pdx-1, or Pdx-1 and insulin antibodies to detect differentiation. The medium containing SP was changed every 24 h. Cells were fixed with 4% paraformaldehyde and then were permeabilized with 0.5 ml of 0.1% Triton X-100. After blocking with 3% BSA, cells were incubated with primary antibody at 4°C overnight. The following primary antibodies were used: mouse anti-CK19 (1:100; Proteintech, Wuhan, China), rabbit anti-Pdx-1(1:100; Proteintech, Wuhan, China) and mouse anti-insulin (1:100; NovusBio, Littleton CO, USA). Cells were then stained with FITC- or Cy3-labeled secondary antibodies (Jackson ImmunoResearch Lab, West Grove, PA at 1:200 dilution). Nuclei were stained with DAPI. Results were visualized with fluorescence microscopy (Nikon Eclipse E600; Nikon Corporation, Tokyo, Japan). The proportion of cells labeling with specific marker in total number of DAPI+ cells was accessed and expressed as the mean percentage of specific differentiation.

### Flow cytometry analysis

To determine the percentage of CK19+ cells in total ductal cells, ductal cells at 3rd passage were stained with CK19. Cells were fixed and permeabilized using Cytofix/Cytoperm system (BD Bioscience, San Diego, CA, USA). After permeabilization, cells were stained with mouse anti Cytokeratin 19 (CK19) antibody for 30 min at 4°C. Then cells were incubated with FITC-labeled secondary antibody for 30 min at room temperature. To detect the differeciation of ductal cells, intracellular staining was performed for insulin production and CK19 expression. After cell permeabilization, cells were stained with mouse anti CK19 and PE-labeled insulin antibodies, following incubation of FITC-labeled secondary antibody. Mouse anti CK19 and PE-labeled anti-insulin antibodies were purchased from Novus Biologicals(Littleton, CO, USA). FITC-labeled anti-mouse secondary antibody was purchased from Santa Cruz Biotechnology (Santa Cruz, CA, USA). Data were analyzed by FACScan flow cytometer (BD Biosciences) using the CellQuest software (BD Biosciences).

### Western blot analysis

Cells were lysed by pre-cooling cell lysis buffer for Western and IP (Beyotime biotechnology, Shanghai, China) supplemented with 1 mM PMSF (Beyotime biotechnology, Shanghai, China) on ice for 15 min and centrifuged at 12,000 × g for 15 min at 4°C. Protein qualification was measured by using a BCA kit (Beyotime biotechnology, Shanghai, China). Forty micrograms total proteins in each lane were electrophoretically separated by 10% SDS-PAGE (Beyotime biotechnology, Shanghai, China) and transferred onto polyvinylidene difluoride (PVDF) membranes (Millipore, Billerica, MA). Membranes were blocked with 3% BSA in TBST buffer (50 mM Tris•HCl, pH 7.4, 150 mM NaCl, 0.1% Tween-20) for 1 h at room temperature. After rinsing with TBST buffer three times, membranes were incubated with primary antibodies overnight at 4°C. The following primary antibodies were used: rabbit anti NK1R (1:1,000; AbSci, Baltimore, MD), rabbit anti GSK-3β (1:1,000; AbSci, Baltimore, MD), rabbit anti β-catenin (1:1,000; Cell Signaling Technology, Boston, MA) and mouse anti β-actin (1:10,000; Sigma, St. Louis, MO). After washing with TBST, membranes were incubated by horseradish peroxidase (HRP)-conjugated anti-rabbit or anti-mouse secondary antibodies for 1 h at room temperature. ECL chemiluminescence solution (Bio-Rad Laboratories, Hercules, CA) was added after rinsing with TBST. Signal intensity of immunoreactive bands was analyzed using Image Lab software (Bio-Rad Laboratories, Hercules, CA) and ImageJ software (Version 1.48v, National Institutes of Health, Bethesda, MD). For the Western blot assay, the optical density of the protein bands was normalized to the density of β-actin band to yield the densitometric ratio value.

### Statistical analysis

Statistical analysis was performed with SPSS version 16.0 (2007; Chicago, IL, USA). Most data are expressed as the mean ± SD, data of Western blot are expressed as the mean ± SEM, and one-way ANOVA with Bonferroni correction for multiple comparisons was used to analyze the differences between the groups. *p* < 0.05 was determined as significance.

## Results

### Generation and identification of rat pancreatic ductal cells

**Figure 1:**
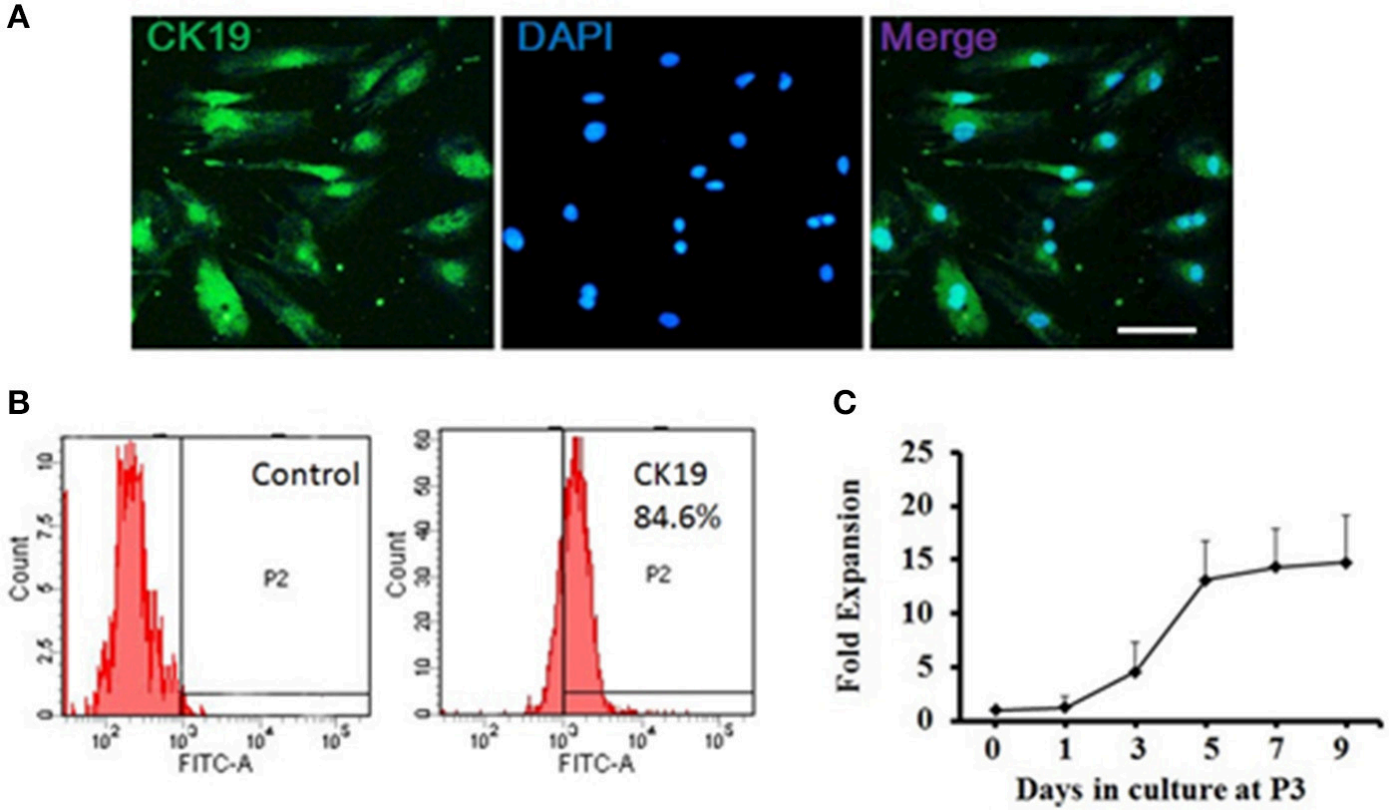
Generation and characterization of adult pancreatic ductal cells *in vitro*. **(A)** Pancreatic ductal cells were cultured and expanded at the third passage (P3) for purification. The P3 cells were immunostained by CK19 (green) antibody and visualized by fluorescent microscopy. Scale bar: 100 μm. **(B)** Flow cytometric analysis of CK19 expression on pancreatic ductal cells. One representative experiment of three is shown. **(C)** Growth curve. Dissociated ductal cells were plated at a density of 1.0 × 10^5^ cells/ml and cultured. At day 1, 3, 5, 7, and 9, the cells were collected and counted by hemocytometer. At least 6 wells were assessed at each time point.

The ductal cells obtained from rat pancreas were expanded at 3rd passage (P3) for purification and characterized phenotypically and molecularly. Cells at P3 were adherent in serum containing medium, and were long, spindle shaped cells in morphology. Majority of the P3 cells were positive for CK19 (Figure 1A), a ductal cell marker. Flow cytometry results showed that more than 80% of single P3 cells were positive for CK19 (Figure 1B), indicating that the isolated cells were ductal cells and qualified for subsequent experiments.

Cell growth was measured at day 1, 3, 5, 7, and 9 to assess the self-renewal capacity of ductal cells. The result showed that the cells expanded rapidly between day 1 and day 5 and plateaued after day 5 (Figure 1C).

### Substance P but not CGRP enhanced self-renewal capacity of ductal cells

**Figure 2:**
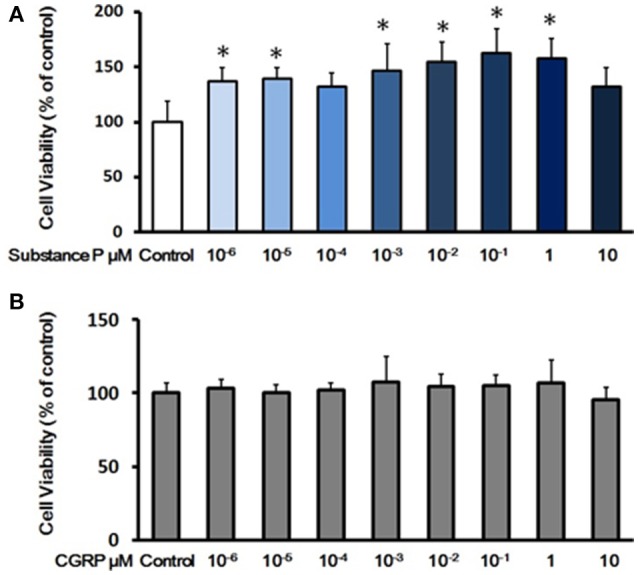
Effects of substance P or CGRP on self-renewal capacity of adult pancreatic ductal cells. **(A)** Cell viability assay with treatment of substance P. Cells were treated with a serial of concentrations of substance P (10^−6^, 10^−5^, 10^−4^, 10^−3^, 10^−2^, 10^−1^, 1, and 10 μM; *n* = 6 wells for each) for 72 h, and the cell viability was measured by CCK-8 assay. **(B)** Cell viability assay with treatment of CGRP. Cells were treated with different concentrations of CGRP (10^−6^, 10^−5^, 10^−4^, 10^−3^, 10^−2^, 10^−1^, 1, and 10 μM; *n* = 6 wells for each) for 72 h, following assayed by CCK-8 test. *P < 0.05, compared with control groups.

Proliferation is tightly linked to self-renewal capabilities of ductal cells. To determine the effects of SP or CGRP on proliferation, P3 ductal cells were treated with series concentrations of SP (10^−6^, 10^−5^, 10^−4^, 10^−3^, 10^−2^, 10^−1^, 1, and 10 μM) or CGRP (10^−6^, 10^−5^, 10^−4^, 10^−3^, 10^−2^, 10^−1^, 1, and 10 μM) in growth medium for 3 days. Cell viability was assessed by CCK-8 test. Compared with control cells, SP (10^−6^, 10^−5^, 10^−3^, 10^−2^, 10^−1^, and 1 μM) treated cells exhibited a stronger proliferation capacity (Figure 2A). However, there was not a significant effect of CGRP on cell viability (Figure 2B), indicating that SP but not CGRP promoted cell proliferation of pancreatic ductal cells.

**Figure 3:**
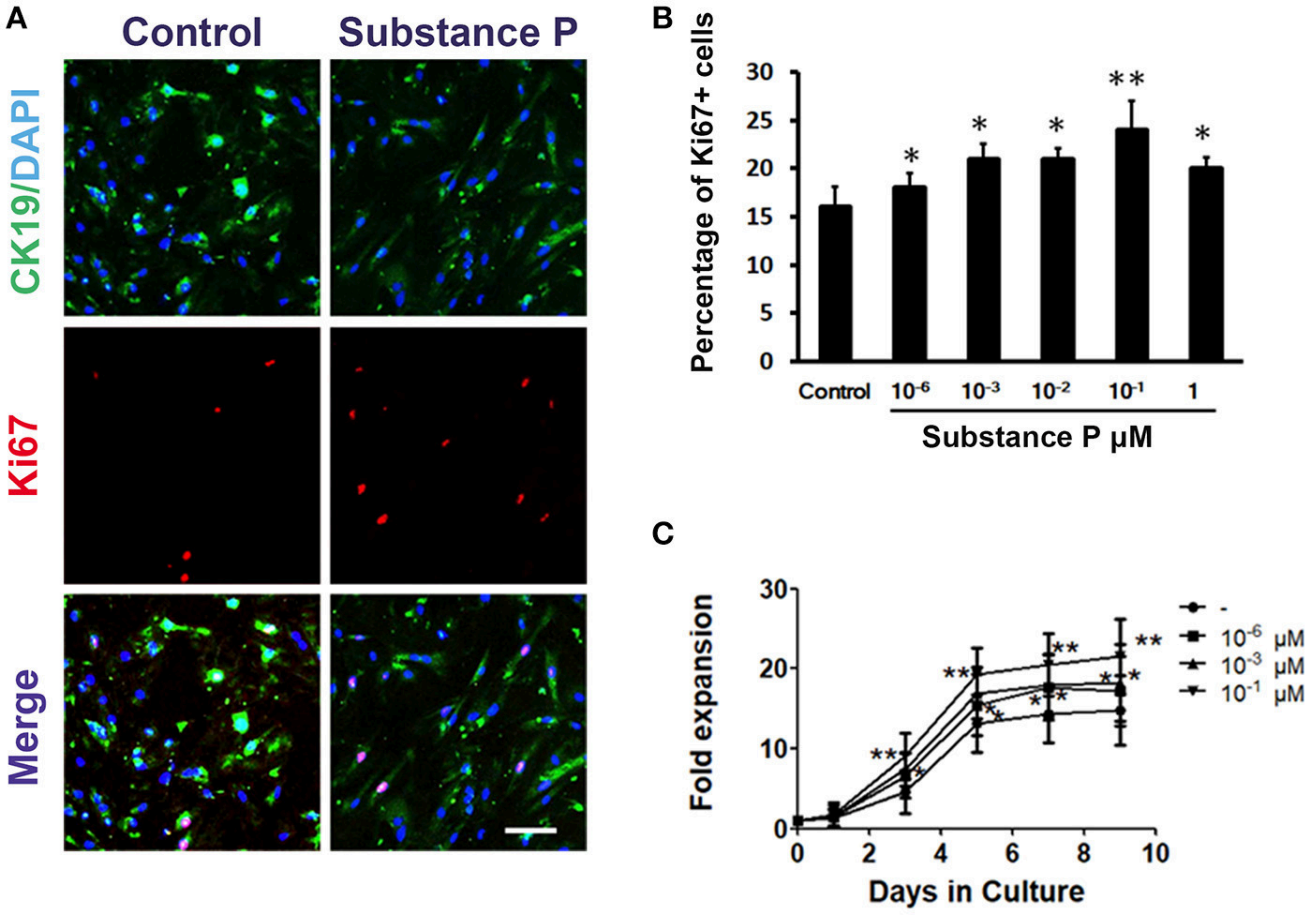
Promotion effects of substance P on proliferation of adult pancreatic ductal cells. **(A)** Ki67 labeling. Cells were treated with substance P (10^−6^, 10^−3^, 10^−2^, 10^−1^, 1, and 10 μM; *n* = 3 wells for each) for 72 h and immunostained by CK19 (green) and Ki67 (red) antibodies. Scale bar: 100 μm. **(B)** Quantitative analysis of Ki67 positive cells. **(C)** Growth curves. Dissociated ductal cells were plated at a density of 1.0 × 10^5^ cells/ml and cultured. Cells were treated without or with substance P (10^−6^, 10^−3^, 10^−1^ μM). At day 1, 3, 5, 7, and 9, the cells were collected and counted by hemocytometer. At least 6 wells were assessed at each time point. *P < 0.05, **P < 0.01, compared with control groups.

To further confirm the effect of SP on ductal cell proliferation, the proliferation of ductal cells was tested with Ki67 (a marker for cell proliferation) immunostaining. After incubation with SP (10^−6^, 10^−3^, 10^−2^, 10^−1^, and 1 μM) for 3 days, ductal cells were immunostained with Ki67 followed by image analysis. As shown in Figures 3A,B, significantly higher levels of Ki67-positive cells were found in SP treated groups compared with control (*P* < 0.05, *P* < 0.05, *P* < 0.05, *P* < 0.01, *P* < 0.05, respectively). Moreover, growth curves of day 1, 3, 5, 7, and 9 were plotted to assay the self-renewal capacity of ductal cells treated with different concentrations of SP (10^−6^, 10^−3^, and 10^−1^ μM). The results indicated that cells incubated with SP expanded more rapidly on day 3, 5, 7, and day 9 (Figure 3C). These findings indicated that SP promoted proliferation of ductal cells.

### Substance P did not induce differentiation of ductal cells into β-cells

**Figure 4:**
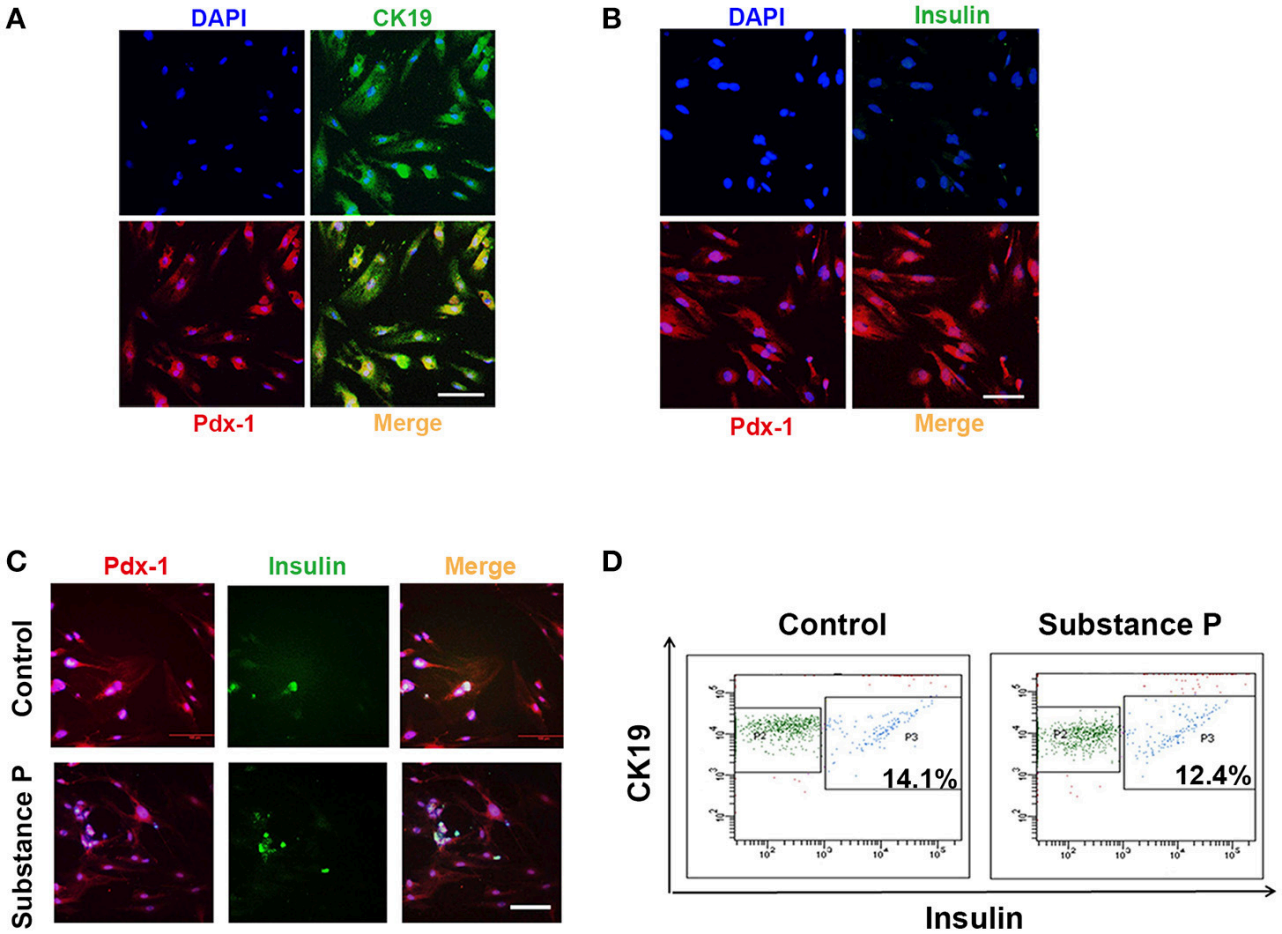
Substance P did not promote differentiation of adult pancreatic ductal cells into Beta-Cells. P3 ductal cells were treated with substance P (10^−6^, 10^−2^, and 10^−1^ μM) for 28 days. Immunostaining was performed to identified the differentiation of ductal cells. **(A)** Cells were Pdx-1 (red) and CK19 (green) positive. Scale bar: 100 μm. **(B)** Cells were Pdx-1 (red) positive but insulin (green) negative. Scale bar: 100 μm. **(C)** After 28 incubation of induction medium (DMEM/F12 plus 2% FBS, 10 mmol/L Niacinamide, 20 ng/ml HGF, 20 μg/L bFGF, and 10 nmol/L exendin-4) with or without substance P (10^−6^, 10^−2^ and 10^−1^ μM) for 28 days, ductal cells were immunostained by Pdx-1 (red) and insulin (green) antibodies. A portion of Pdx-1 positive cells expressed insulin. Scale bar: 100 μm. **(D)** Flow cytometric analysis of CK19 and insulin expression on pancreatic ductal cells. Data was compared with isotype-matched controls. One representative experiment of three is shown.

Above results showed ductal cells were sensitive to SP, and cell proliferation was promoted at a concentration of 10^−6^ μM. Next, we tested whether SP further promotes differentiation on ductal cells. To determine the effects of SP on differentiation of ductal cells, cells were treated with serial doses of SP (10^−6^, 10^−^1, and 1 μM) for 28 days. In substance P treated groups, cells positive for CK19 and Pdx-1 were negative for insulin (Figures 4A,B), as verified by triple immunostaining, indicating that SP dose not directly induce differentiation of adult pancreatic ductal cells into β-cells.

Further, cell culture medium was replaced by insulin-produced induction medium with or without SP (10^−6^, 10^−1^, and 1 μM). After 28 days, ductal cells developed into insulin-produced cells (insulin+) (Figure 4C). Flow cytometric analysis showed that SP did not promote ductal cells differentiated into greater numbers of insulin-produced cells (*P* > 0.05, *P* > 0.05, respectively, Figure 4D) as compared with induction medium control group. All these results indicated that SP did not affect the differentiation potential of ductal cells into β-cells.

### Blockade of SP-induced stimulation of proliferation with NK-1 receptor antagonist l-703,606

**Figure 5:**
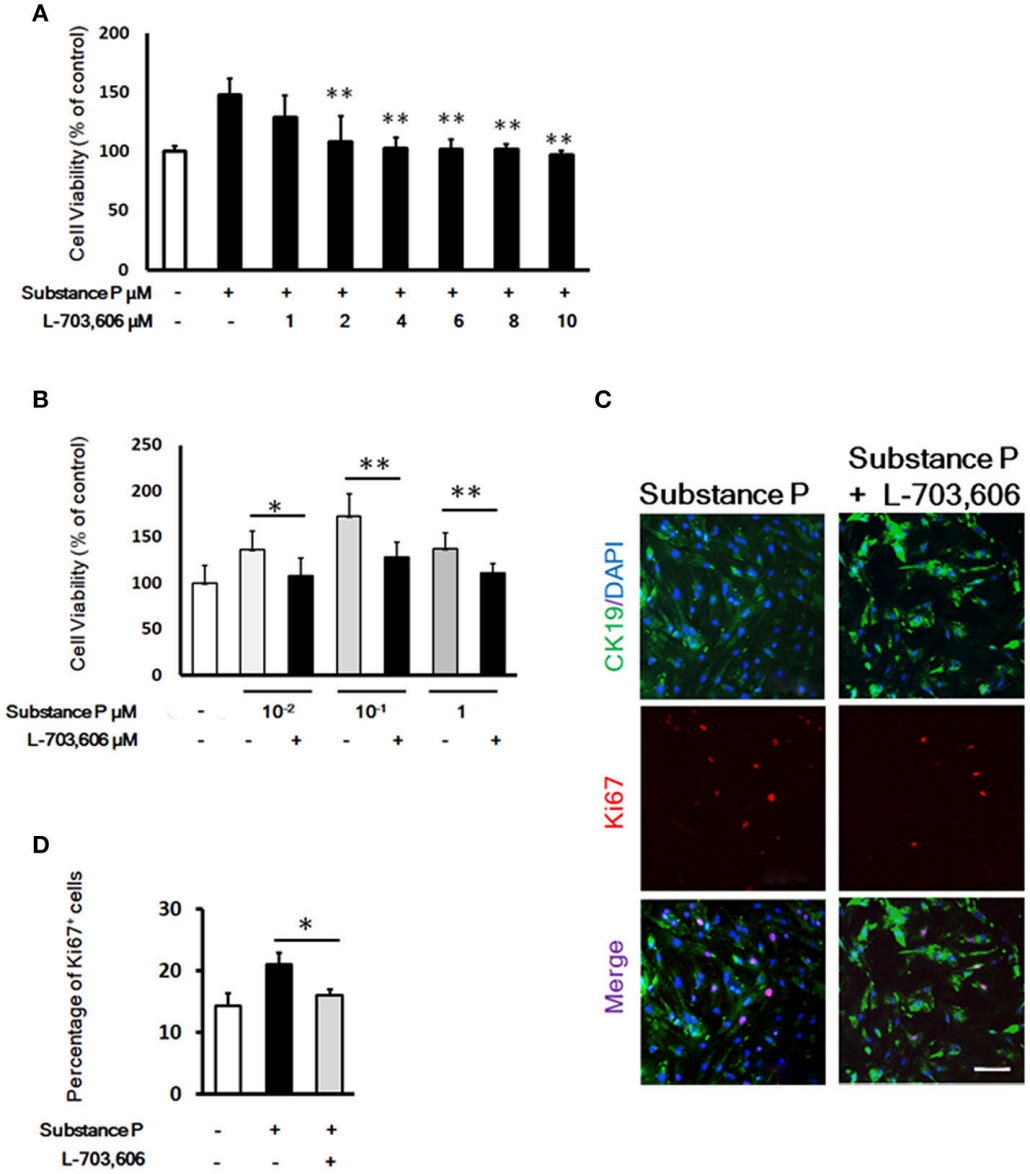
L-703,606 blocked the promotion effects of substance P on proliferation of pancreatic ductal cells. **(A)**. Effect of L-703,606 on proliferative effects of substance P in pancreatic ductal cells. Cells were treated with SP (10^−1^ μM) plus different concentrations of L-703,606 (1, 2, 4, 6, 8, 10 μM) for 72 h, and the cell viability was measured by CCK-8 assay. **(B)** Cell viability assay. Cells were treated with different concentrations of substance P (10^−2^, 10^−1^, and 1 μM) with or without L-703,606 (2 μM) for 72 h, and the cell viability was measured by CCK-8 assay. **(C)** Ki67 labeling. Cells were treated with substance P (10^−1^ μM) with or without L-703,606 (2 μM) for 72 h. Cells were immunostained by CK19 (green) and Ki67 (red) antibodies. Scale bar: 100 μm. **(D)** Quantitative analysis of Ki67 positive cells. *P < 0.05, **P < 0.01, as compared with substance P treated groups.

To evaluate the influence of NK-1 receptor (NK1R) antagonist L-703,606 on the effects of SP on duct cells, cells were incubated with SP (10^−1^ μM) plus L-703,606 (1, 2, 4, 6, 8, 10 μM) for 72 h. After 72 h incubation, cell viability was measured by CCK8 assay. Exposure of SP-treated cells to L-703,606 (2, 4, 6, 8, 10 μM) significantly inhibited the cell proliferation induced by SP (*P* < 0.01, *P* < 0.01, *P* < 0.01, *P* < 0.01, *P* < 0.01, respectively, Figure 5A). The dose of 2 μM of L-703,606 was used to block the stimulatory effects of SP on duct cells in further experiments.

To investigate whether SP could mediate ductal cell proliferation via NK-1 receptor, we added NK1R antagonist L-703,606 (2 μM) into the culture medium containing different doses of SP (10^−2^, 10^−1^, and 1 μM). In L-703,606 plus SP-treated groups, L-703,606 treatment decreased cell viability of ductal cells compared with SP-treated groups (percentage of control without SP or L-703,606, 108.02 ± 19.65 vs. 136.10 ± 20.59 in 10^−2^ μM, *P* < 0.05; 128.50 ± 16.33 vs. 172.81 ± 24.15 in 10^−1^ μM, *P* < 0.01; 110.95 ± 10.78 vs. 137.14 ± 17.06 in 1 μM, *P* < 0.01; Figure 5B).

Ki67 immunostaining was performed to verify the inhibition of L-703,606 on proliferation effects of SP on ductal cells. Compared with control groups, the SP-treated cells showed a significantly higher number of cells positive for Ki67 (21.34 ± 1.89% vs. 16.17 ± 1.04%, *P* < 0.05, Figures 5C,D). Compared with SP-treated groups (10^−1^ μM), L-703,606 treatment decreased the number of Ki67 positive cells (16.17 ± 1.04% vs. 21.34 ± 1.89%, *P* < 0.05, Figures 5C, D). These results mean that SP plays its role in proliferation of ductal cell via NK-1 receptor.

### Substance P promotes NK1R expression, decreases GSK-3β and increased β-catenin in ductal cells

**Figure 6:**
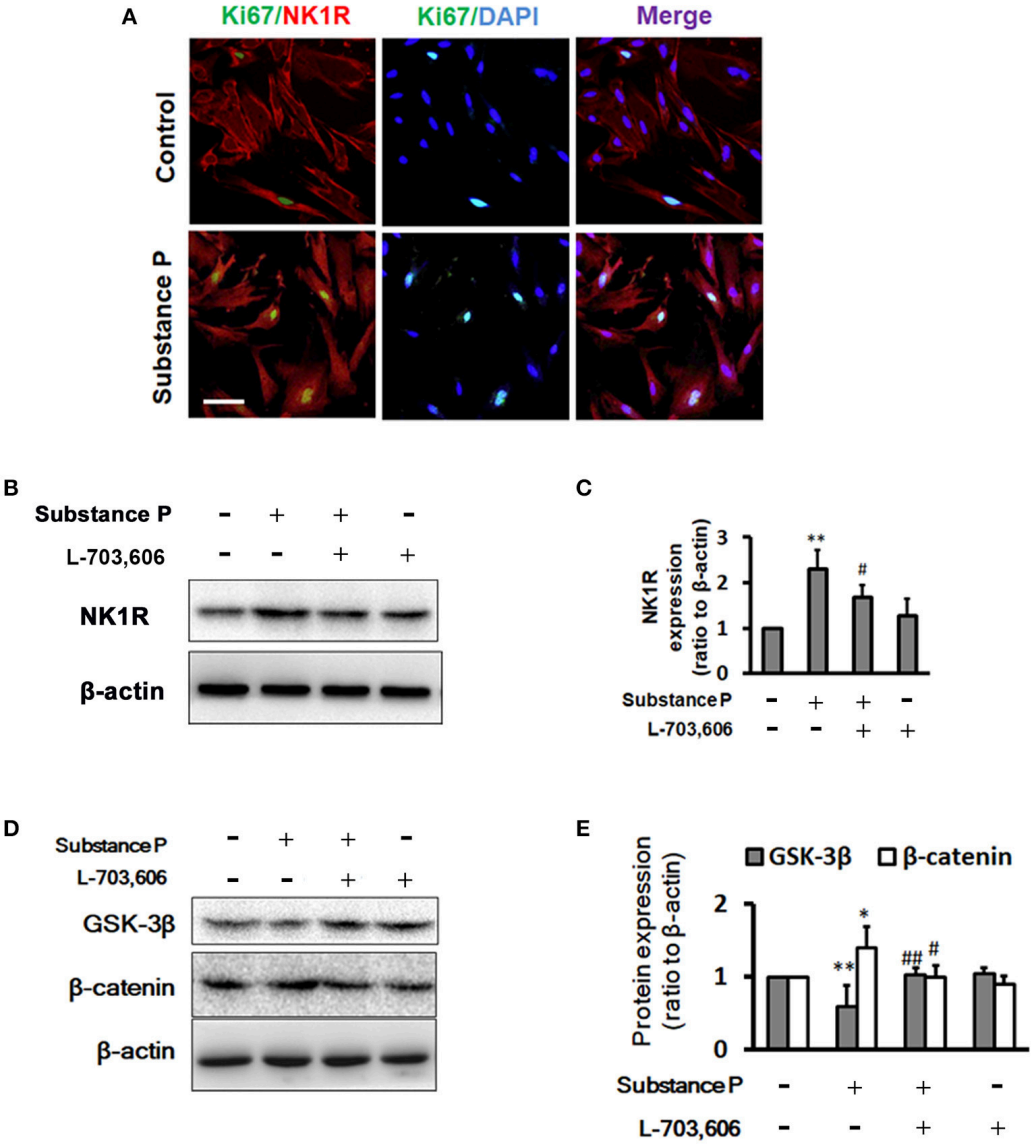
L-703,606 attenuated the effects of substance P on NK1R, GSK-3β, and β-catenin expression. **(A)** Cells were NK1R (red) and Ki67 (green) positive in cells treated without (control cells) or with substance P (10^−1^ μM). Scale bar: 100 μm. **(B)** Western Blot images of NK1R. Cells were incubated with or without substance P (10^−1^ μM) and/or L-703,606 (2 μM) for 72 h. Cell extracts were analyzed for the expression of NK1R. **(C)**. Quantitative analysis of NK1R. Substance P increased NK1R expression, whereas L-703,606 blocked the effects of substance P on NK1R. **(D)** Western Blot images of GSK-3β and β-catenin. Cells were incubated with or without substance P (10^−1^ μM) and/or L-703,606 (2 μM) for 72 h. GSK-3β and β-catenin expression were measured in cell extracts by Western blot. **(E)** Quantitative analysis of GSK-3β and β-catenin. Substance P reduced GSK-3β levels and increased β-catenin levels, whereas L-703,606 blocked the effects of substance P on GSK-3β and β-catenin. Densitometric ratios are the mean ± SEM of three experiments. **P < 0.01, *P < 0.05 as compared with control groups. ^##^*P* < 0.01, ^#^*P* < 0.05 as compared with substance P treated groups.

We performed double immunostaining of NK1R and ki67 expression on ductal cells. Similar to Garland's finding (Garland et al., 1994), we found that NK1R was expressed in cytoplasm after SP treatment (Figure 6A). To investigate whether SP affect NK1R, and GSK-3β/β-catenin pathway, cells were treated with SP (10^−1^ μM), SP (10^−1^ μM)+L-703,606 (2 μM) and L-703,606 (2 μM) alone for 72 h, total protein was harvested and Western blot analysis was performed to test expression of NK1R, GSK-3β and β-catenin (Figures 6B,D). Quantitative analysis revealed that NK1R protein levels were significantly increased in SP treated cells as compared with control groups (Figure 6C, *P* < 0.01), but L-703,606 treatment significantly decreased NK1R levels (Figure 6C, *P* < 0.05). Quantitative analysis of GSK-3β and β-catenin protein showed that SP treatment significantly decreased GSK-3β levels (Figure 6E, *P* < 0.01) and significantly increased β-catenin levels (Figure 6E, *P* < 0.01) compared with control group, whereas L-703,606 treatment decreased these effects of SP on ductal cells (SP+L-703,606 group vs. SP group, *P* < 0.05, *P* < 0.05, respectively).

## Disccusion

In the present study, we demonstrated that SP but not CGRP promoted cell viability and proliferation of ductal cells derived from rat pancreas, which is inhibited by the NK1 receptor antagonist. SP did not exert the effects of differentiation toward β-cell of these cells. Our experiments also revealed that SP treatment enhanced NK1R expression, reduced GSK-3β levels and increased β-catenin levels. However, treatment with NK1R inhibitor L-703,606 attenuated the effects of SP on NK1R, GSK-3β and β-catenin levels. Collectively, these findings suggest that SP promotes pancreatic duct cell proliferation via NK1R, and the Wnt/β-catenin pathway is involved in the mechanisms of SP-induced proliferation of ductal cells.

SP and CGRP have been showed to be capable to promote proliferation in some other tissues. SP has been reported to promote proliferation of adult neural progenitor cells (NPCs) (Park et al., 2007), cord blood CD34+ hematopoietic stem cells (Shahrokhi et al., 2010), and fibroblast-like cells derived from bile duct (Tian et al., 2014). CGRP stimulates cell proliferation and inhibits cell apoptosis of bone-marrow MSCs in short term culture, and promotes osteogenetic differentiation in long term culture (Xu and Jiang, 2014; Liang et al., 2015). It has been reported that CGRP overexpressed rat adipose-derived stem cells (ADSCs) exerted more potency of proliferation, neurosphere formation (Yang et al., 2014), and osteoblastic differentiation (Fang et al., 2013). Moreover, CGRP and SP treatment promoted human skin keratinocyte proliferation (Shi et al., 2013). Besides promoting proliferation, SP has also been shown to stimulate differentiation of mesenchymal stem cells (MSCs) into osteoblastic cells through neurokinin (NK)-1 receptor (Sun et al., 2010).

Our results showed that SP stimulated proliferation of pancreatic ductal cells and pancreatic ductal cells were very sensitive to SP on proliferation. Opolka et al. (2012) reported that SP at the concentration of 10^−4^ μM promoted proliferation of murine chondrocytes. SP at 10^−3^ μM induces pro-proliferation effects on fibroblast-like cells from bile duct and hematopoietic stem cells (Shahrokhi et al., 2010; Tian et al., 2014). In bone marrow-derived mesenchymal stem cell-like cells and bone marrow stromal stem cells, 10^−2^ μM SP is needed to promote cell proliferation (Mei et al., 2013; Dubon and Park, 2015; Liu et al., 2016). SP concentration as high as 10^−1^ μM is needed in ARPE-19 cells, a human retinal pigmented epithelial (RPE) cell line (Baek et al., 2016), and neural stem/progenitor cells derived from spinal cord to show its effect (Kim et al., 2015). In bone marrow mesenchymal stem cells (MSCs), SP at as low as 10^−6^ μM enhances osteoblast differentiation of MSCs (Wang et al., 2009) and protects against apoptosis induced by serum deprivation (Fu et al., 2015). Our results indicated that SP at as low as 10^−6^ μM had pro-proliferation effect on ductal cells. Thus, pancreatic ductal cells are among the most sensitive cells to SP, similar to MSCs.

The actions of SP are mediated by three G protein–coupled receptors, NK-1, NK-2, and NK-3 receptors; among these, the NK-1 receptor (NK1R) has the highest affinity for SP (Takeda et al., 1991). In Kim's (Kim et al., 2015) and Park's (Park et al., 2007) reports, NK1R inhibitor L-703,606 (1–10 μM) decreased SP-induced proliferation of spinal cord-derived neural stem/progenitor cells (SC-NSPCs). We examined the inhibitory effect of L-703,606 (1–10 μM) on SP-stimulated cell proliferation. Our results indicated that L-703,606 at dose of 2–10 μM effectively blocked the proliferative effect of SP. Our results showed that L-703,606 inhibited the proliferation effects of SP on pancreatic ductal cells, indicating the proliferation stimulating effect of SP on ductal cells through NK1R. Notably, several studies are consistent with our results. Glaser et al. (2011) reported that knockout of the NK1R reduced cholangiocyte proliferation in bile duct-ligated mice, indicating a specific mitotic effect of SP on bile ductal cells. In Liu's study (Liu et al., 2016), they demonstrated that SP enhanced proliferation of bone marrow mesenchymal stem cell derived osteoblasts (BMSC-OB), and this effect would be inhibited by adding NK1R antagonist. In has been shown that pretreatment with the NK1R antagonist reduced the effect of SP on proliferation of ARPE-19 cells (Baek et al., 2016).

It has been reported that SP at concentration of 1 μM increased NK1R mRNA expression in pancreatic acinar cells (Koh et al., 2012). In our study, we performed double immunostaining of NK1R and ki67 expression on ductal cells. The images showed that the ductal cells had stronger NK1R immune staining in SP-treated cells by compared with control cells. Garland et al. (1994) reported that NK1R was located in intracellular vesicles, rather than aggregated in the plasma membrane in epithelial cells after incubation with SP. Similar to Garland's finding, we found that NK1R was expressed in cytoplasm after SP treatment. Consistent with the results of staining, our results of Western blotting showed that the level of NK1R was significantly higher in SP treated group, compared with control group.

It is interesting that CGRP did not show any effect on proliferation of ductal cells. SP and CGRP are two important neurotransmitters released from primary sensory fibers of dorsal root ganglia. CGRP and SP are colocalized and co-released from these fibers (Gibbins et al., 1985). Besides primary sensory fibers of dorsal root ganglia, SP was also from another important source which is intrapancreatic ganglia. Neurons in pancreatic ganglia innervate islets as well as pancreatic ducts (Sha et al., 2001; Love et al., 2007). Our study (Shen et al., 2016) revealed that intrapancreatic ganglia contain SP neurons and contribute around 50% of SP in pancreas. Our results suggested that SP released from primary sensory fiber and fibers of pancreatic ganglia stimulate proliferation of pancreatic ducts. Pancreatic ganglia don't contain any CGRP neurons (our unpublished data) and all CGRP fibers appear coming from primary sensory neurons (Russell et al., 2014). Thus, via release of CGRP, primary sensory fibers might uniquely modulate activity other than proliferation of pancreatic duct.

Pancreatic ductal cells play as pancreatic stem cells that exhibit stem cell properties, including proliferation and differentiation potential. Even though SP showed pro-proliferation activity, we did not find SP exert any promoting effect on the ductal cells differentiating toward β-cells. Our results showed that SP did not induce pancreatic ductal cells differentiate into insulin-positive cells directly, although the ductal cells all expressed Pdx-1, a pancreatic progenitor cell marker (Murtaugh, 2007). Further, in insulin-produced cell induction medium, SP did not promote pancreatic ductal cells differentiating into greater numbers of insulin-produced cells either.

Activation of Wnt signaling has been shown to be essential for proliferation of multiple cells (Mah et al., 2016; Arrigoni et al., 2018; Majidinia et al., 2018). Glycogen synthase kinase-3β (GSK-3β), a key molecule of the Wnt pathway, induces phosphorylation of β-catenin, causing the degradation of β-catenin. GSK-3β activity is inhibited following activation of the Wnt pathway, stimulating the accumulation of β-catenin in the cytoplasm, and β-catenin transferred into the nucleus, serving as a transcription factor to induce the activation of downstream target genes (Wang et al., 2018). We propose that SP regulates ductal cell proliferation via activation of Wnt pathway. Our results of western blot showed that SP treatment enhanced NK1R expression, decreased GSK-3β levels and increased β-catenin levels. Moreover, L-703,606, a NK1R antagonist, decreased NK1R expression and attenuated the effects of SP on GSK-3β and β-catenin levels, indicating that SP may promote proliferation of ductal cells via augmenting NK1R and GSK-3β/β-catenin signaling.

Our study was designed to understand the possible effects of neuropeptides released by primary sensory nerve fibers on pancreatic ductal cells. However, we found that only SP promoted the proliferation of pancreatic ductal cells. Moreover, SP did not induce or promote the differentiation of ductal cells into β-cells. The onset of diabetes is related to the lack of SP. Razavi et al. (2006) reported that reduced release of substance P initiates immune stress response of islet β-cells, leading to inflammation of islet cells and invasion of lymphocytes. On top of that, the ductal cells in the pancreas have stem cell properties and are one of the sources of β-cell neogenesis (Xu et al., 2008; Bonner-Weir et al., 2010). The proliferative effect of SP on ductal cells may explain another possible pathological course in the developmental stage of diabetes. The stabilization of SP levels might indicate that the pancreatic endogenous β-cell pool is abundant and is one of the conditions for maintaining β-cell homeostasis. Therefore, the lack of SP might be an indicator of the initial of diabetes. In addition, SP can also be used as a drug candidate for the treatment of diabetes.

In summary, SP but not CGRP stimulates the proliferation of pancreatic ductal cells. SP does not induce or promote the differentiation of ductal cells toward β-cells. Moreover, SP may promote proliferation of pancreatic ductal cells through enhancing NK1R expression, decreasing GSK-3β and increasing β-catenin levels.

## Informed consent

Informed consent was obtained from all individual participants included in the study.

## Author contributions

NZ, DG, YL, and SJ participated as investigators and reviewed, edited, and approved of the final version of the manuscript. LS initiated the study of neuropeptides substance P and CGRP in diabetes, is the guarantor of this work, had full access to all the data in the study and takes responsibility for the integrity of the data and the accuracy of the data analysis. NZ and LS wrote the manuscript.

### Conflict of interest statement

The authors declare that the research was conducted in the absence of any commercial or financial relationships that could be construed as a potential conflict of interest.
